# The dual PI3K/mTOR inhibitor NVP-BEZ235 inhibits proliferation and induces apoptosis of burkitt lymphoma cells

**DOI:** 10.1186/s12935-015-0213-1

**Published:** 2015-06-24

**Authors:** Chuntuan Li, Pengliang Xin, Huifang Xiao, Yan Zheng, Yuanling Huang, Xiongpeng Zhu

**Affiliations:** Department of Haematology, First Hospital of Quanzhou Affiliated to Fujian Medical University, 248 East Street, Licheng District, Quanzhou, 362000 Fujian Province China

**Keywords:** Burkitt lymphoma, Phosphatidylinositol 3-kinase/Akt/mammalian target of rapamycin pathway, NVP-BEZ235, Cell proliferation, Apoptosis

## Abstract

**Background:**

Phosphatidylinositol 3-kinase/Akt/mammalian target of rapamycin (PI3K/Akt/mTOR) pathway is a therapy target of cancer. We aimed to confirm the effect of dual PI3K/mTOR inhibitor NVP-BEZ235 on cell proliferation and apoptosis in Burkitt lymphoma (BL) cells.

**Methods:**

Two human BL cell lines, CA46 and RAJI were used in this study. The proliferation of BL cells was detected by manganese tricarbonyl transfer (MTT) assay. Cell cycle and apoptosis assay were examined by flow cytometric analysis. The phosphorylation levels of AKT (Thr308), AKT (Ser473), and RPS6 were evaluated by western blot analysis.

**Results:**

NVP-BEZ235 significantly inhibited the proliferation of BL cells (CA46 and RAJI) and the inhibition effect was time and dose-dependent. Cell cycle analysis indicated that the cells (CA46 and RAJI) were mostly arrested in G1/G0 phase. Cell apoptosis assay showed that the late apoptotic cells were significantly increased after 72 h treatment by 100 nmol/L of NVP-BEZ235. In addition, results also found that NVP-BEZ235 reduced the phosphorylation levels of AKT (Thr308), AKT (Ser473), and PRS6 in BL cells (CA46 and RAJI). Moreover, this inhibition effect on phosphorylation was dose-dependent.

**Conclusions:**

NVP-BEZ235 effectively inhibited cell proliferation by G0/G1 cell-cycle arrest and induced apoptosis through deregulating PI3K/Akt/mTOR pathway in BL cells.

## Background

Burkitt lymphoma (BL), as a highly aggressive non-Hodgkin lymphoma, drives from germinal center (GC) B cells [1, 2]. There are three recognized clinical variants based on the WHO classification: endemic (eBL, found predominantly in equatorial Africa), sporadic (sBL, the predominant type found in non-malarial areas), and associated with immunodeficiency (including human immunodeficiency virus–associated and post-transplantation lymphoproliferative disorder after solid organ transplantation) [1, 3, 4]. Based on recent reports and statistics, BL is the most common form of non-Hodgkin lymphoma in children [5, 6] and the incidence is higher in males than in females [7–9]. Currently, chemotherapy remains the main treatment modality for BL. However, the acquired chemoresistance remains a challenging issue and reduces the possibility of effective salvage and cure [10]. Meanwhile, the clinical outcome is still poor in patients with over 40 years old [2, 11]. Therefore, a novel and effective treatments are needed to enhance the efficacy of chemotherapy and improve clinical outcomes in the treatment of BL patients.

The phosphatidylinositol 3-kinase/Akt/mammalian target of rapamycin (PI3K/Akt/mTOR) pathway deregulation is a common event in human cancer and associated with the tumor cell proliferation, growth and apoptosis [12, 13]. Currently, this pathway has become a favorable therapy target of cancer [13–15], including BL [16, 17]. However, there is still no feasible and effective drug targeting this pathway in the clinical treatment of BL. Meanwhile, it has been reported that the deregulation of PI3K/Akt/mTOR pathway can leading to chemoresistance in BL [18]. Thus, it is important to find a novel drug targeting PI3K/Akt/mTOR pathway for treating BL.

NVP-BEZ235 is a dual inhibitor of PI3K and mTOR. It is a synthetic compound belonging to the class of imidazoquinolines, and inhibits PI3K and mTOR catalytic activity by competitively binding to the ATP-binding cleft [19]. Previous studies have reported its inhibition in tumor cell proliferation and growth as well as promotion in apoptosis in many other cancers [20–22]. Meanwhile, Shortt et al. [23] reported that NVP-BEZ235 induced apoptosis of BL cells was associated with the PI3K/Akt/mTOR pathway in MYC-driven BL cells. However, it is unknown that whether this effect of NVP-BEZ235 still exist in BL cells without MYC-driven. Moreover, Shortt et al. [23] also showed that the BEZ235-induced apoptosis occurred independently of p53. Thus, we have performed this study using two BL cell lines (CA46 and RAJI, which all have mutant p53) to further assess and confirm the effects of NVP-BEZ235 on BL cells.

## Materials and methods

### Cell lines and reagents

Two human BL cell lines, CA46 and RAJI, were purchased from KeyGEN Biotech (NanJing, China), and cultured in RPMI 1640 medium which contained 10 % newborn calf serum (Gibco, Waltham, MA, USA) in a humidified 37 °C incubator with 5 % CO_2_.

NVP-BEZ235 was purchased from Selleckchem (Houston, TX, USA) and dissolved in dimethylsulfoxide (DMSO) to a concentration of 10 mmol/L. Before experiment, NVP-BEZ235 was stored at −20 °C. In the following experiments, it would be further diluted to an appropriate final concentration.

### Cell proliferation assay

Cells from two cell lines (CA46 and RAJI) were respectively seeded in different 96-well plates with 10 % newborn calf serum at a density of 1 × 10^4^ cells/well. The cells were respectively treated with NVP-BEZ235 at different concentrations (1, 10, 50, 100, 500 and 1000 nmol/L) for 24, 48, and 72 h. Meanwhile, the cells incubated with equal volume of DMSO instead of NVP-BEZ235 were used as control. After incubation period, 3-(4, 5-dimethylthiazolyl-2)-2, 5-diphenyltetrazolium bromide (MTT, Amresco, OH, USA) was immediately added to the wells at a final concentration of 20 μmol/L and the cells were incubated for 4 h. Subsequently, the cells were collected through centrifugation. The supernatant was discarded and 150 μl DMSO was used to suspend the cells in each well. Finally, measurement of absorbance at 490 nm was performed using an automatic multi-well spectrophotometer (Bio-Tek Instruments, Vermont, USA). The inhibition rate was calculated based on the formula: Inhibition rate (%) = (1 - Absorbance of wells treated with NVP-BEZ235 /Absorbance of control) × 100.

### Cell cycle analysis

Cells from two cell lines were respectively seeded in different 6-well plates with 10 % newborn calf serum at a density of 1 × 10^5^ cells/ml. The cells were respectively treated with NVP-BEZ235 at concentrations of 10 and 100 nmol/L for 48 h. Meanwhile, the cells incubated with equal volume of DMSO instead of NVP-BEZ235 were used as control. After incubation period, cells were harvested through centrifugation and washed twice with ice-cold phosphate-buffered saline (PBS). Afterwards, the cells were fixed using 1 ml ice-cold PBS and 3 ml ethanol at −20 °C overnight. After fixation, the cells were washed twice with ice-cold PBS again, and then incubated with 30 μg/ml of propidium iodide (PI, Sigma-Aldrich, St Louis, MO) and 40 μg/ml of Rnase (Sigma-Aldrich, St Louis, MO) for 30 min at room temperature in a dark room. Before analysis by the FACSCalibur cytometer (BD Biosciences, San Jose, CA), the cells were separated through a 200-mesh nylon filter. Data were analyzed using the Modfit LT software (BD Biosciences, San Jose, CA).

### Cell apoptosis assay

BL cells were seeded in 6-well plates with 10 % newborn calf serum at a density of 1 × 10^5^ cells/ml. The cells were respectively treated with 100 nmol/L of NVP-BEZ235 for 48 h and 72 h. Meanwhile, the cells incubated with equal volume of DMSO instead of NVP-BEZ235 were used as control. After treatment, cells were washed twice with ice-cold PBS. Subsequently, apoptosis was assayed using the FITC Annexin V Apoptosis Detection Kit I (BD Biosciences, San Jose, CA, USA), following the manufacturer’s instructions. The cells were assessed using flow cytometric analysis. Viable cells were FITC Annexin V and PI -negative cells; cells that were FITC-Annexin V-positive and PI-negative were considered as being in early apoptosis; whereas necrotic cells were FITC-annexin-V negative/low-PI positive; cells that were both FITC-Annexin V- and PI-positive were considered as being in late apoptosis;

### Western blot analysis

In the PI3K/Akt/mTOR pathway, AKT is the central key component of this pathway and activated through phosphorylation at Ser473 and Thr308. Moreover, ribosomal protein S6 (RPS6) would be phosphorylated in the downstream of mTOR signaling [13]. Thus, we detected the phosphorylation levels of these proteins through western blot analysis to assess if NVP-BEZ235 could affect the PI3K/Akt/mTOR pathway in BL cells.

BL cells were seeded in 6-well plates with 10 % newborn calf serum at a density of 1 × 10^6^ cells/ml. Meanwhile, the cells were respectively treated with different concentrations of NVP-BEZ235 (1, 10, 100, and 200 nmol/L) for 0, 3, 6,12, 24, and 48 h. The cells incubated with equal volume of DMSO instead of NVP-BEZ235 were used as control. Western blotting was performed as described previously [24]. Briefly, after washing twice by PBS, cells were lysed with RIPA (Radio Immunoprecipitation Assay) lysis buffer (Solarbio, Beijing, China), which contained 1 mmol/L PMSF (Phenylmethanesulfonyl fluoride). The protein concentrations of the cell lysates were determined using the Enhanced BCA Protein Assay kit (Beyotime, Haimen, China). Proteins were separated by sodium dodecyl sulfate-polyacrylamide (SDS) gel electrophoresis and then transferred to nylon membranes. The membranes were blocked in TBS-T buffer solution containing 5 % non-fat dry milk at 4 °C overnight. Immunoblotting was performed using rabbit polyclonal antibodies against AKT (1:100 dilution), phosphorylated Akt (Ser473 or Thr308, both 1:100 dilution), RPS6 (1:100 dilution), phosphorylated RPS6 (1:100 dilution), and β-actin (1:1000 dilution), respectively. Blots were probed with horseradish peroxidase–conjugated secondary antibody (anti-rabbit) and developed using enhanced chemiluminescence reagent (Amersham Biosciences, Piscataway, NJ). All antibodies were purchased from Bioss (BeiJing, China). Band density was imaged and the levels of protein expression quantified using Molecular Imager VersaDoc MP 4000 system (Bio-Rad) and normalized to the β-actin levels. The phosphorylation levels of proteins were assessed based on the ratios of phosphorylated proteins to the corresponding total proteins.

### Statistical analysis

The data were presented as means ± SD. Comparison between groups were performed using a Non-parametric test or one-way ANOVA. A value of P < 0.05 was considered statistically significant. Statistical analyses were performed using SPSS 17.0 software (Chicago, USA).

## Results

### NVP-BEZ235 inhibited BL cell proliferation

**Fig. 1:**
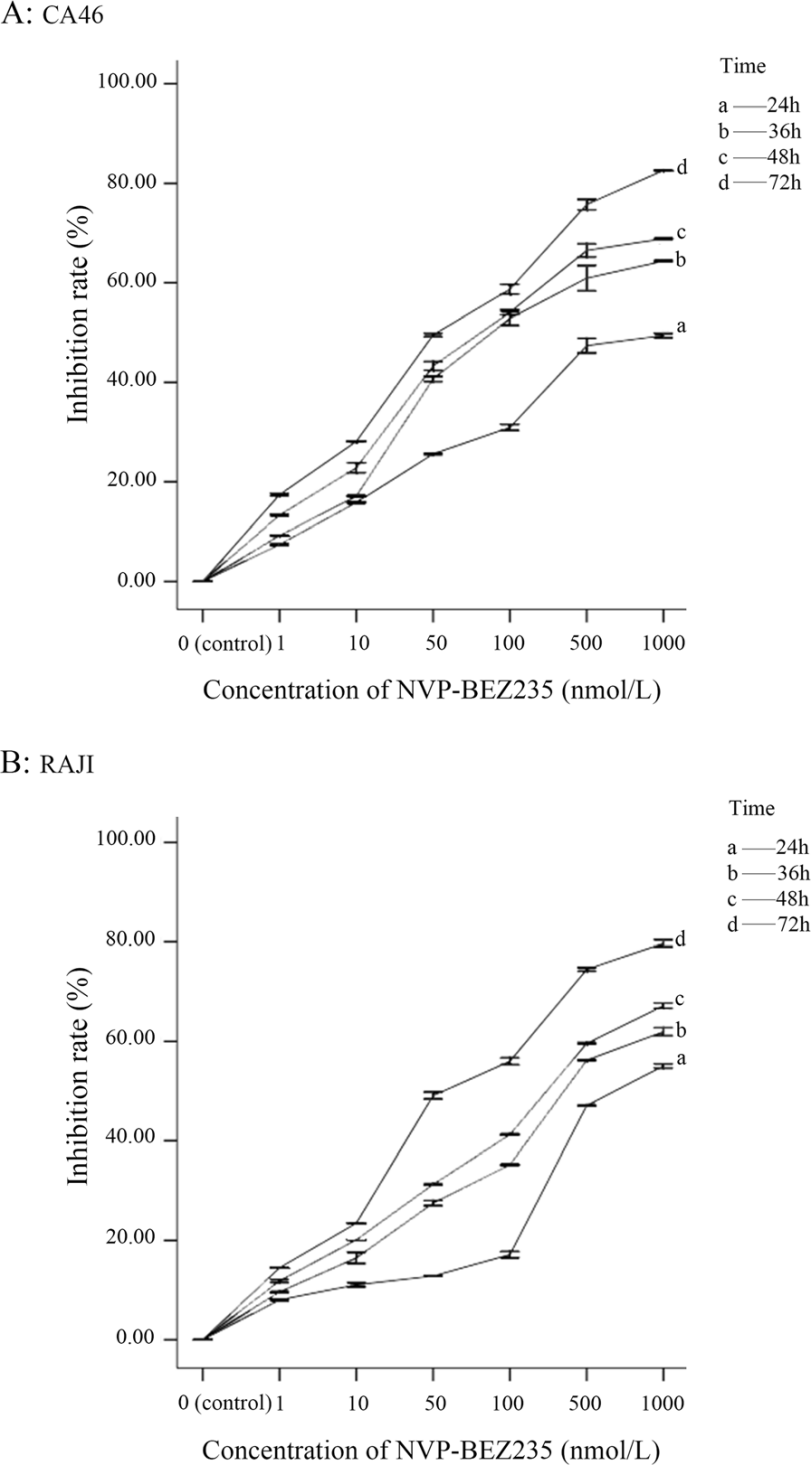
NVP-BEZ235 inhibited the proliferation of Burkitt lymphoma cells. **a** CA46; **b** RAJI

As shown in Fig. 1, the inhibition rates of two BL cell lines were significantly increased with the increasing incubation time and concentration of NVP-BEZ235 (P < 0.05), indicating that NVP-BEZ235 could inhibit the proliferation of BL cells and this antiproliferative effect was time and dose-dependent.

### G0/G1 phase arrest induced by NVP-BEZ235

**Fig. 2:**
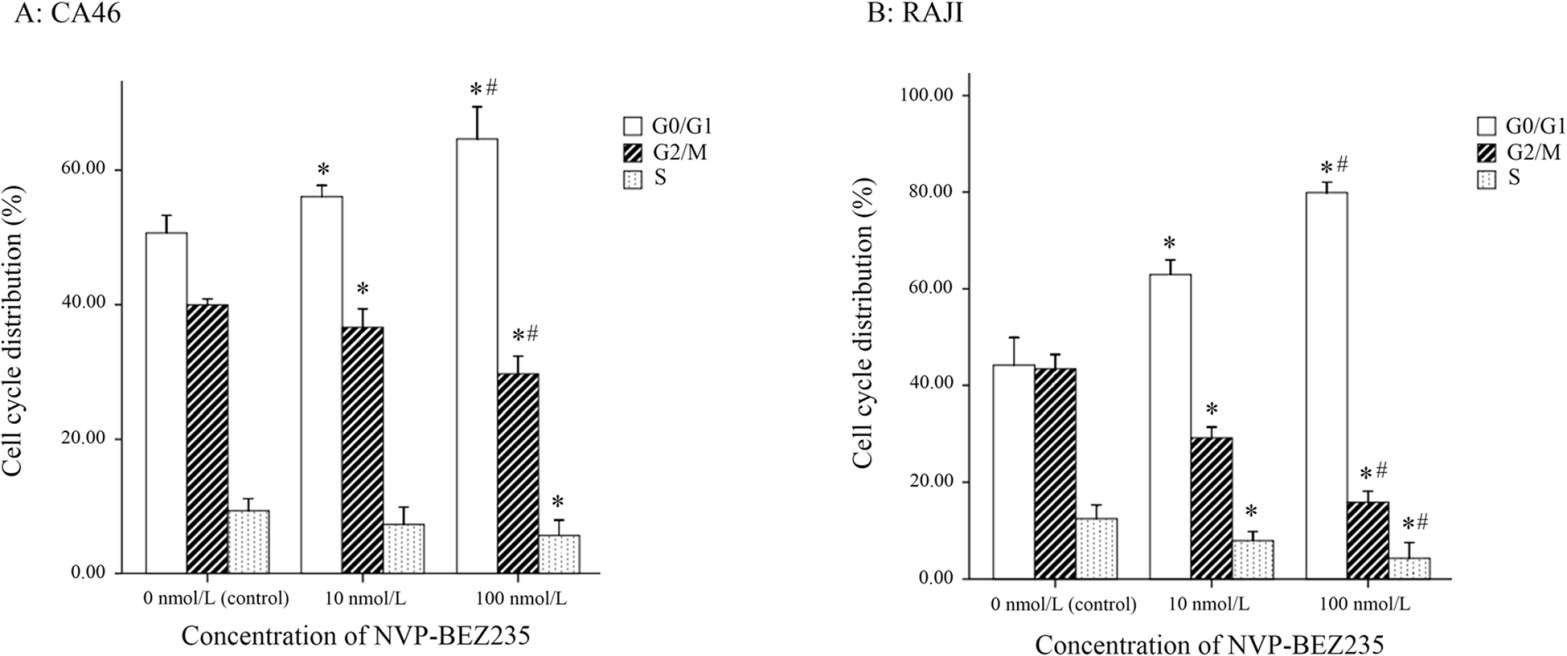
NVP-BEZ235 blocked the cell cycle of Burkitt lymphoma cells at the G1/G0 phase. **a** CA46; **b** RAJI. ^*^ *P* < 0.05, compared with control; ^#^ *P* < 0.05, compared with the cells treated with 10 nmol/L NVP-BEZ235

**Fig. 3:**
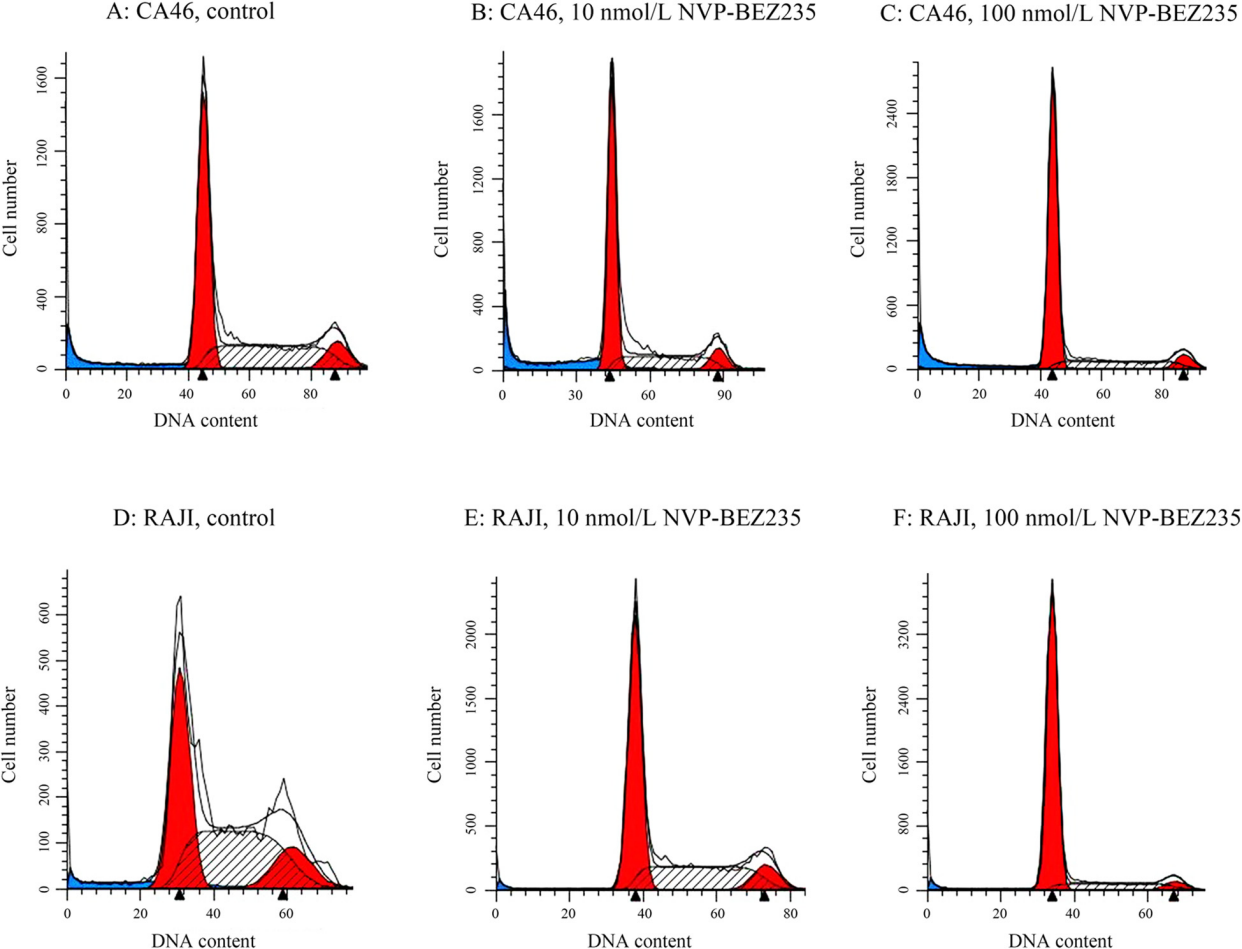
Cell cycle distribution was analyzed by flow cytometry

NVP-BEZ235 induced cell cycle arrest in G1/G0 phase in both BL cell lines (Figs. 2 and 3). Compared with control, cells in the G1/G0 phase were significantly increased (P < 0.05) and in G2/M and S phases were significantly reduced after 48 h treatment by10 nmol/L or 100 nmol/L of NVP-BEZ235 (P < 0.05). Moreover, compared with cells treated with 10 nmol/L NVP-BEZ235, there were significantly more cells in G1/G0 phase when cells were treated with 100 nmol/L NVP-BEZ235 (P < 0.05).

### Effect of NVP-BEZ235 on cell Apoptosis

**Fig. 4:**
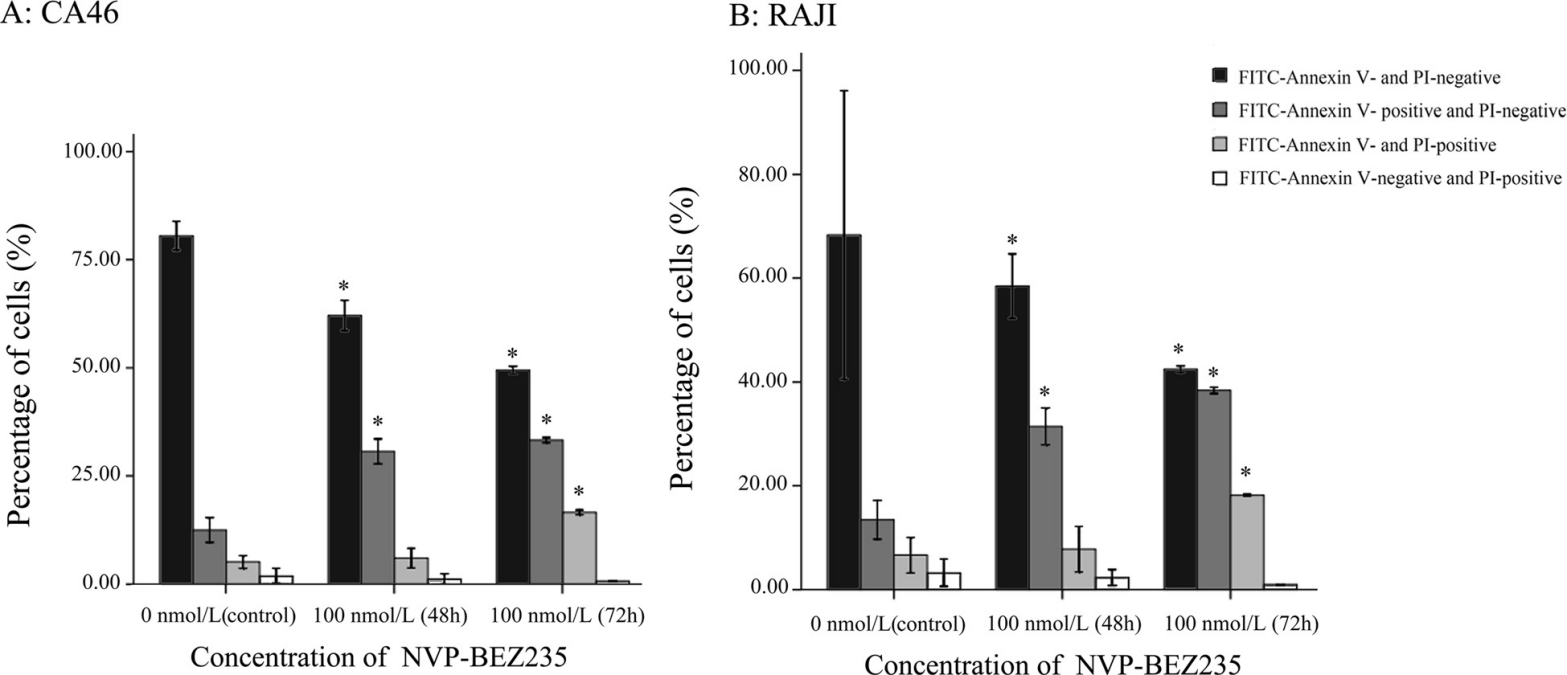
NVP-BEZ235 induced apoptosis in Burkitt lymphoma cells. **a** CA46; **b** RAJI. ^*^ *P* < 0.05, compared with control

**Fig. 5:**
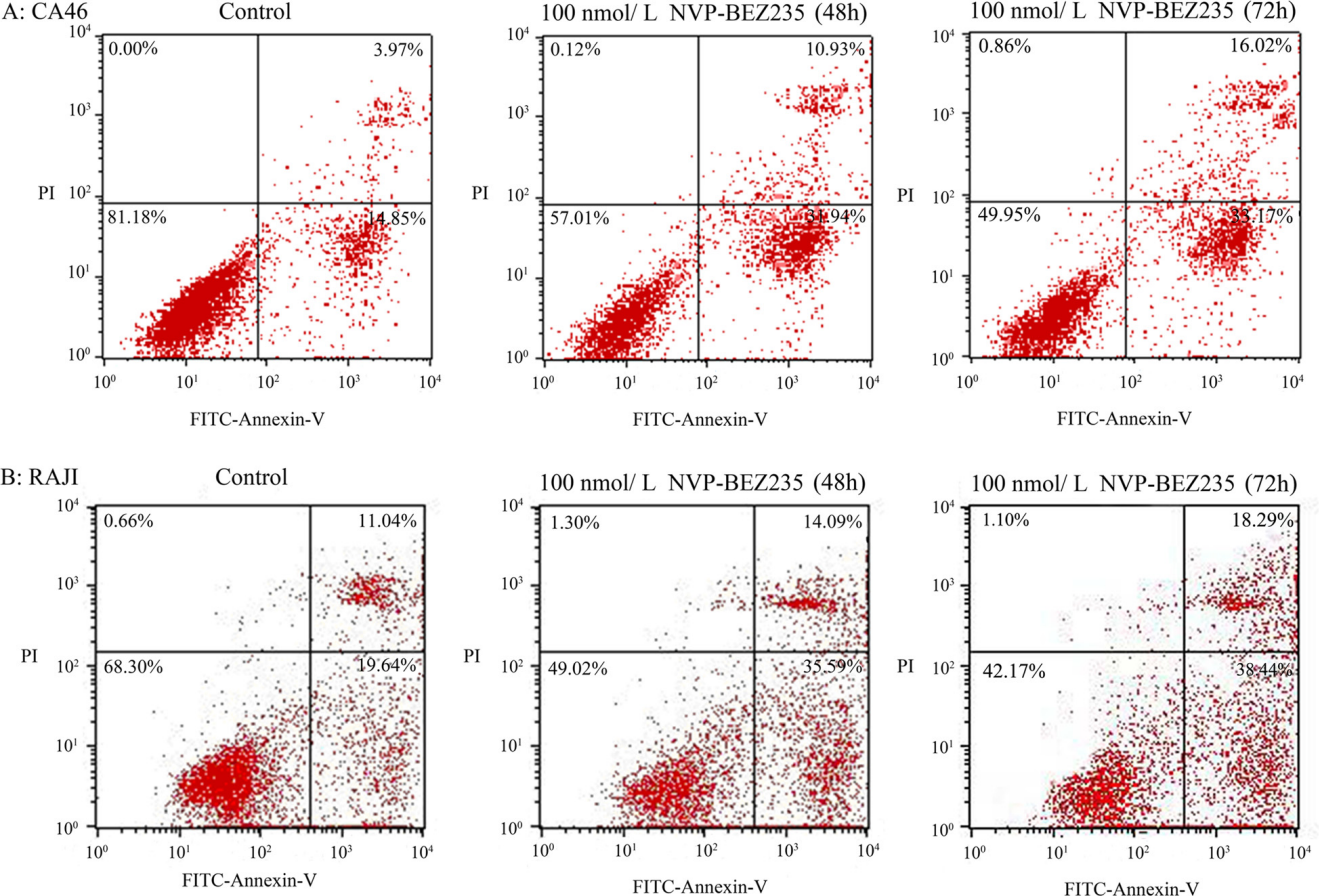
Apoptosis was detected by flow cytometry using Annexin-V and propidium iodide staining methods

Compared with control, the viable cells (FITC Annexin V and PI negative) were significantly reduced and the early apoptotic cells (FITC-Annexin V-positive and PI-negative) were significantly increased by the treatment of 100 nmol/L of NVP-BEZ23 for 48 h and 72 h (P < 0.05). Moreover, although 48 h treatment by 100 nmol/L of NVP-BEZ235 did not cause the significant increase of late apoptotic cells (FITC Annexin V and PI positive) (P > 0.05), the late apoptotic cells were significantly increased after 72 h incubation of 100 nmol/L of NVP-BEZ235 (*P* < 0.05, Figs. 4 and 5).

### Effect of NVP-BEZ235 on PI3K/AKT/mTOR pathway in BL cells

**Fig. 6:**
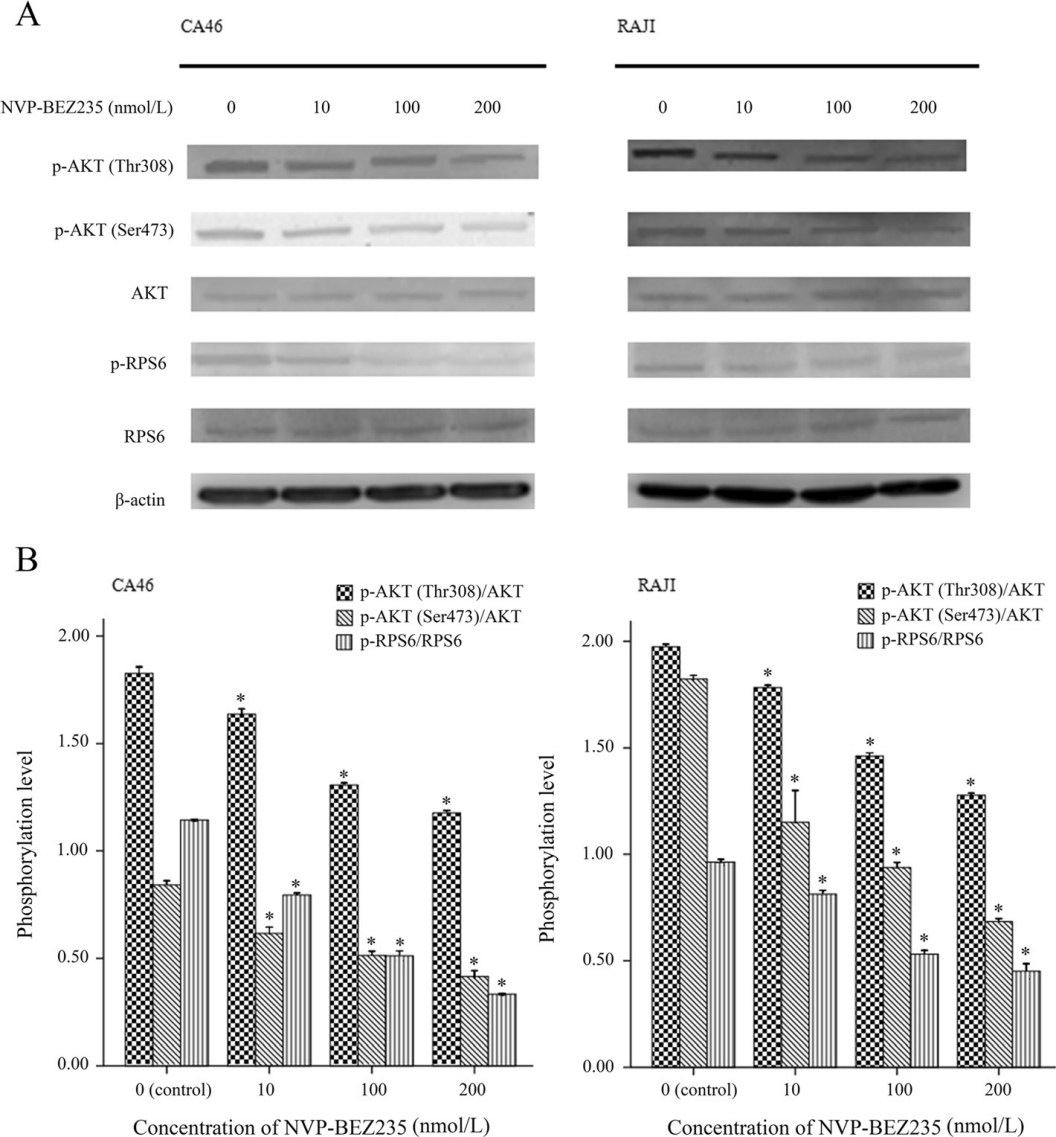
Dose dependent inhibition effect of NVP-BEZ235 on the phosphorylation levels of AKT (Thr308), AKT (Ser473), and PRS6 in Burkitt lymphoma cells. **a** results of western blot; **b** phosphorylation levels of AKT (Thr308), AKT (Ser473), and PRS6 in cells treated with NVP-BEZ235 at concentrations of 0, 10, 100, and 200 nmol/L for 12 h. ^*^ *P* < 0.05, compared with control

**Fig. 7:**
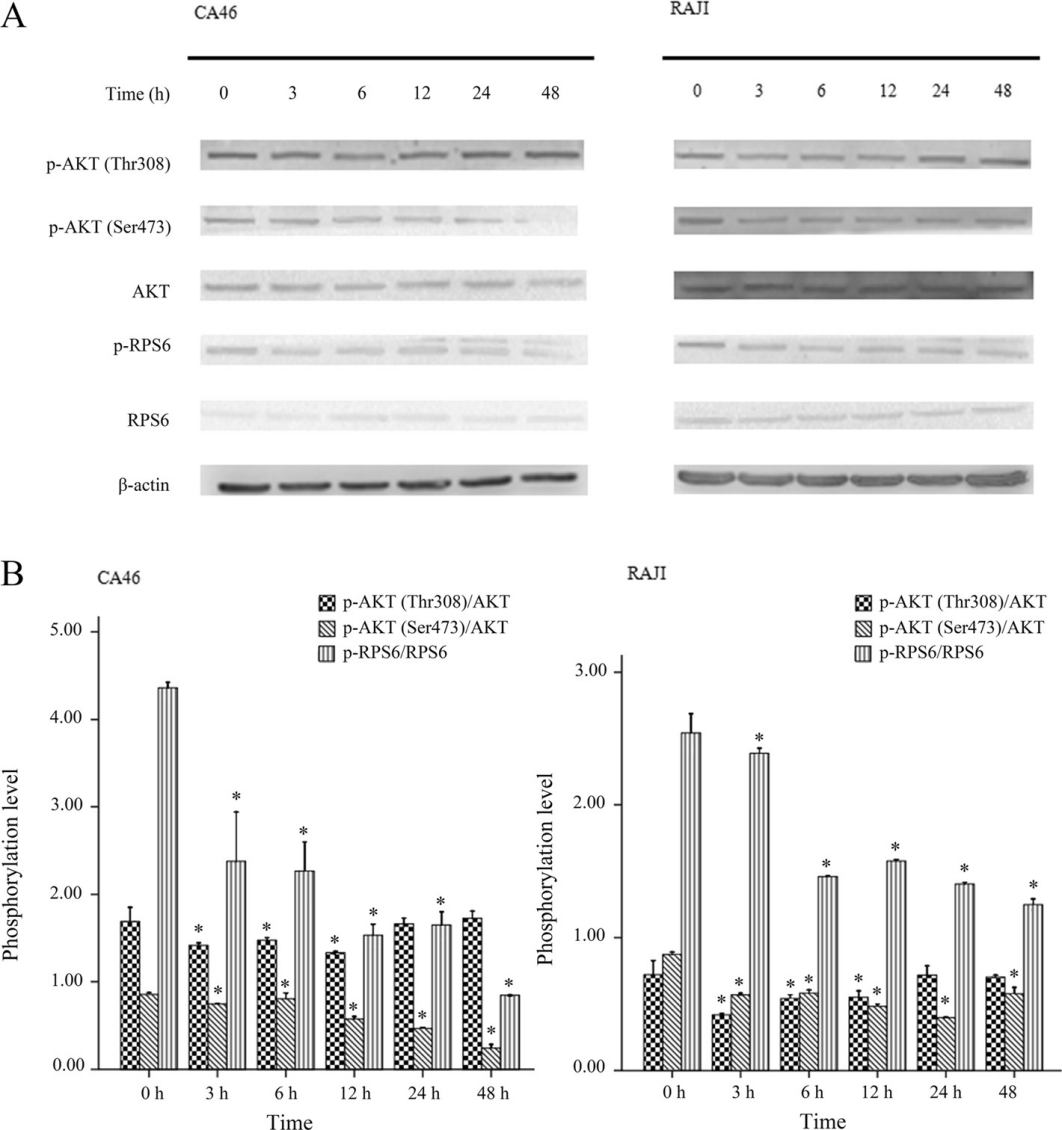
Phosphorylation levels of AKT (Thr308), AKT (Ser473), and PRS6 were significantly changed in the cells treated with 100 nmol/L NVP-BEZ235 for 0 h, 3 h, 6 h, 12 h, 24 h, and 48 h. **a** results of western blot; **b** phosphorylation levels of AKT (Thr308), AKT (Ser473), and PRS6 in cells treated with 100 nmol/L NVP-BEZ235 for 0 h, 3 h, 6 h, 12 h, 24 h, and 48 h. ^*^ *P* < 0.05, compared with control

Figures 6 and 7 showed the western blotting results. The phosphorylation levels of AKT (Thr308), AKT (Ser473), and PRS6 were modestly inhibited by NVP-BEZ235 in both CA46 and RAJI cells (*P* < 0.05). Moreover, the inhibition effect was significantly enhanced with the increasing dose of NVP-BEZ235, indicating this inhibition effect was dose-dependent (*P* < 0.05, Fig. 6b). In addition, the phosphorylation levels of AKT (Ser473) and PRS6 were also modestly decreased after the cells were incubated with 100 nmol/L of NVP-BEZ235 for 3, 6, 12, 24, and 48 h. Meanwhile, NVP-BEZ235 also modestly inhibited the phosphorylation of AKT (Thr308) with the incubation time of 3, 6, 12 h (*P* < 0.05). However, the phosphorylation level of AKT (Thr308) recovered to baseline after the cells were treated by 100 nmol/L of NVP-BEZ235 for 24 and 48 h (*P* > 0.05). Results indicated that there was no obvious time–dependent inhibition effect of NVP-BEZ235 on the phosphorylation of AKT (Thr308), AKT (Ser473), and PRS6 (Fig. 7b).

## Discussion

New therapeutic strategies are necessary for BL, because of the resistance of chemotherapy and poor outcomes. The PI3K/Akt/mTOR pathway is a therapy target of cancer. In this study, we found that the NVP-BEZ235 (a dual inhibitor of PI3K and mTOR) effectively inhibited cell proliferation and prompted cell apoptosis in two BL cell lines. Also, the cells were mostly arrested in G1/G0 phase by NVP-BEZ235 in BL cells. Moreover, the inhibition effect of NVP-BEZ235 on cell proliferation was time and dose-dependent. In addition, the phosphorylation levels of AKT (Thr308), AKT (Ser473), and PRS6 were also modestly changed by NVP-BEZ235. The dose-dependent inhibition effect of NVP-BEZ235 was also found on phosphorylation of AKT (Thr308), AKT (Ser473), and PRS6. However, this inhibition effect was not time-dependent.

Similar with other cancers (such as endometrial carcinomas [25], human pancreatic cancer [26], thyroid cancer [27] and osteosarcoma [28]), NVP-BEZ235 also suppresses cell proliferation by inducing G0/G1 cell-cycle arrest in BL cells. Meanwhile, significantly decreased phosphorylation of AKT (Thr308), AKT (Ser473), and PRS6 in BL cells treated by NVP-BEZ235, indicated that the PI3K/Akt/mTOR pathway was deregulated by NVP-BEZ235 in BL cells. It was reported that BL cells (P493-6) could be arrested in G0/G1 after the expression of myc was switched off, which was associated with the inhibition of a set of cell cycle activators (cyclin D2, cyclin E and Cdk4) [29]. Moreover, the expression of Cyclin D was found to be associated with activation of Akt, which was depended on the phosphorylation of AKT (Thr308) and AKT (Ser473), in PI3K/Akt/mTOR pathway [30]. In addition, it was reported that the PI3K/Akt/mTOR pathway involved Akt-mediated phosphorylation of FoxO transcription factors, which was required by the Myc-induced proliferation and transformation [31]. Meanwhile, down-regulation of Cyclin D involves the cell cycle arrest induced by FoxO Transcription Factors [32]. Therefore, the G0/G1 cell-cycle arrest induced by NVP-BEZ235 in this study might be caused by the inhibition of PI3K/Akt/mTOR pathway, which could suppress Myc-induced proliferation by decreasing the expression of Cyclin D and phosphorylation of FoxO transcription factors. Besides, the dose-dependent effect demonstrated that high dose of NVP-BEZ235 could significantly inhibit the PI3K/Akt/mTOR pathway than low dose of NVP-BEZ235. The most appropriate application dose of NVP-BEZ235 need to be further investigated.

In this study, apoptosis of BL cells was also induced by NVP-BEZ235. Considering the mutant of p53 in the two cell lines (CA46 and RAJI), the independently of p53 in the NVP-BEZ235 induced apoptosis of BL cells was confirmed, which was in line with the results of Shortt et al. [23]. However, the mechanism for NVP-BEZ235 induced apoptosis of BL cells still need more studies to explore. Previous studies have reported that the S6 kinase (S6K) play roles in different mechanisms of apoptosis [33–35]. In PI3K/Akt/mTOR pathway, activation of mTOR results in the phosphorylation of numerous substrates, including the phosphorylations of S6 kinase (S6K) by mTORC1 [36]. Then the PRS6, as the substrate of S6K, would be phosphorylated [37]. Moreover, it was reported that the PRS6 was also significantly associated with apoptosis (such as gamma irradiation-induced apoptosis, and cisplatin-induced apoptosis) [38, 39]. Thus, we speculated that the effect of NVP-BEZ235 on apoptosis of BL cells may be associated with the phosphorylation of PRS6 and SK6 may play important roles in this effect.

However, some limitation should be noted in this study. Firstly, we have found the inhibition effect of NVP-BEZ235 on PI3K/Akt/mTOR pathway which was associated with chemoresistance of BL cells [18]. Nevertheless, we have not concerned on the changes of chemoresistance by NVP-BEZ235 in BL cells. Further studies were required to investigate it for further exploring the clinical value of NVP-BEZ235. Secondly, the time dependent effect of NVP-BEZ235 on inhibiting cell proliferation was significant, but there was no obvious time–dependent inhibition effect of NVP-BEZ235 on the phosphorylation of AKT (Thr308), AKT (Ser473), and PRS6. Especially for the phosphorylation level of AKT (Thr308), it recovered to baseline after the cells were treated by NVP-BEZ235 for 24 h and 48 h. Hence, the time-dependent effect of NVP-BEZ235 on BL cells demands more investigations and evidences. In addition, because the FACS staining was just used for assessing the effect of NVP-BEZ235 on cell cycle in this study, so the FACS staining after incubating cells with the drug for 72 h was not performed to verify the effect of NVP-BEZ235 on apoptosis. Although NVP-BEZ235 induced apoptosis of BL cells were found by the Annexin V and PI staining after 72 h treatment by 100 nmol/L of NVP-BEZ235, further studies were required to confirm the results of this study.

## Conclusions

In conclusion, NVP-BEZ235 might effectively inhibit BL cells proliferation by G0/G1 cell-cycle arrest through deregulating the PI3K/Akt/mTOR pathway, which was associated with the suppression of Myc-induced proliferation. Moreover, the BL cells apoptosis may be induced by NVP-BEZ235 through decreasing the phosphorylation of PRS6. This study further confirmed the effect of NVP-BEZ235 on BL cells and provided evidence for the clinical application of NVP-BEZ235 on the treatment of BL.

## Abbreviations

BL: Burkitt lymphoma
GC: Germinal center
MTT: Manganese tricarbonyl transfer
PBS: Phosphate-buffered saline
PI3K/Akt/mTOR: Phosphatidylinositol 3-kinase/Akt/mammalian target of rapamycin
SDS: Sodium dodecyl sulfate-polyacrylamide
